# Hyperoxia Improves Hemodynamic Status During Head-up Tilt Testing in Healthy Volunteers: A Randomized Study

**DOI:** 10.1097/MD.0000000000002876

**Published:** 2016-03-03

**Authors:** Julien Fromonot, Guillaume Chaumet, Olivier Gavarry, Jean-Claude Rostain, Michel Lucciano, Fabrice Joulia, Michele Brignole, Jean-Claude Deharo, Regis Guieu, Alain Boussuges

**Affiliations:** From the UMR-MD2, Dysoxie Suractivité, Institut de Recherche Biomédicale des Armées (IRBA) & Aix-Marseille Université, Faculté de Médecine Nord, Marseille, France (JF, GC, J-CR, FJ, J-CD, RG, AB); Laboratoire HandiBio EA 4322, Université du Sud Toulon Var, La Garde, France (OG); Laboratoire de biomécanique appliquée, Aix Marseille Université, Faculté de Médecine Nord, Marseille, France (ML); and Department of Cardiology, Arrhythmologic Centre, Ospedali del Tigullio, Lavagna, Italy (MB).

## Abstract

Head-up tilt test is useful for exploring neurally mediated syncope. Adenosine is an ATP derivative implicated in cardiovascular disturbances that occur during head-up tilt test. The aim of the present study was to investigate the impact of hyperoxia on adenosine plasma level and on hemodynamic changes induced by head-up tilt testing.

Seventeen healthy male volunteers (mean age 35 ± 11 years) were included in the study. The experiment consisted of 2 head-up tilt tests, 1 session with subjects breathing, through a mask, medical air (FiO_2_ = 21%) and 1 session with administration of pure oxygen (FiO_2_ = 100%) in double-blind manner. Investigations included continuous monitoring of hemodynamic data and measurement of plasma adenosine levels.

No presyncope or syncope was found in 15 of the 17 volunteers. In these subjects, a slight decrease in systolic blood pressure was recorded during orthostatic stress performed under medical air exposure. In contrast, hyperoxia led to increased systolic blood pressure during orthostatic stress when compared with medical air. Furthermore, mean adenosine plasma levels decreased during hyperoxic exposure before (0.31 ± 0.08 μM) and during head-up tilt test (0.33 ± 0.09 μM) when compared with baseline (0.6 ± 0.1 μM). Adenosine plasma level was unchanged during medical air exposure at rest (0.6 ± 0.1 μM), and slightly decreased during orthostatic stress. In 2 volunteers, the head-up tilt test induced a loss of consciousness when breathing air. In these subjects, adenosine plasma level increased during orthostatic stress. In contrast, during hyperoxic exposure, the head-up tilt test did not induce presyncope or syncope. In these 2 volunteers, biological study demonstrated a decrease in adenosine plasma level at both baseline and during orthostatic stress for hyperoxic exposure compared with medical air.

These results suggest that hyperoxia was able to increase blood pressure during head-up tilt test via a decrease in plasma adenosine concentration. Our results also suggest that adenosine receptor antagonists are worth trying in neurocardiogenic syncope.

## INTRODUCTION

Adenosine is an endogenous nucleoside that strongly impacts the cardiovascular system via 4 G-coupled membrane receptors, namely A_1_ R, A_2A_ R, A_2B_ R, and A_3_ R, depending on their pharmacological properties.^1^ Activation of A_1_ R leads mostly to bradycardia, whereas activation of A_2A_ R leads mostly to vasodilation.^1^

Head-up tilt test (HUT) is a useful tool for exploring neurally mediated syncope.^2,3^ Because of its cardiovascular effects, adenosine is the humoral factor most likely involved in cardiovascular disturbances observed during HUT. Indeed, strong adenosine release is associated with hemodynamic disturbances during HUT.^4^ Furthermore, there is a positive correlation between the increase in adenosine plasma level (APL) and the rapidity of onset of tilt-induced syncope.^4^ Lastly, an overexpression of adenosine A_2A_ R has been reported in patients with unexplained syncope and a positive HUT.^5–7^

Adenosine is released in the extracellular spaces during hypoxia or inflammatory process.^8^ An increase in APL has been associated with bradycardia and loss of consciousness during experimental hypoxia induced by breath-holding.^9^ Altitude-induced hypoxia alters cardiovascular response to orthostatism,^10^ and experimental hypoxia favored vasovagal reaction via a vasodilation mechanism.^11^ Conversely, hyperoxia is associated with a decrease in extracellular adenosine level.^12^ Consequently, partial pressure of oxygen (PO_2_) impacts cardiovascular status, partly via the modulation of extracellular adenosine level. It was shown that hyperoxic chemoreflex sensitivity is impaired in patients with neurocardiogenic syncope,^13^ but the influence of hyperoxia on HUT had never been evaluated. The aim of this study was, therefore, to assess the effects of oxygen administration on both the hemodynamic status and the APL during HUT in healthy volunteers.

According to previous knowledge, our hypothesis was that hyperoxic exposure increased blood pressure (BP) during HUT when compared with medical air exposure, via its vasoconstrictor effect and a decrease in APL.

## METHODS

Seventeen male volunteers (mean age 35 ± 11 y) were investigated to assess the cardiovascular impact of hyperoxia during abrupt changes in position. In women, the fluctuations in hormones during the menstrual cycle lead to variations in central arterial compliance and sympathetic neural responses to orthostatic stress.^14,15^ To exclude this potential bias, only men were included in the study.

The study was performed in a double-blind manner, and the protocol was approved by the local ethics committee (Aix Marseille University, n° 2010-A011113–36). Informed consent was obtained from volunteers, and the study was carried out in accordance with the WHO code of ethics (Declaration of Helsinki).

**FIGURE 1:**
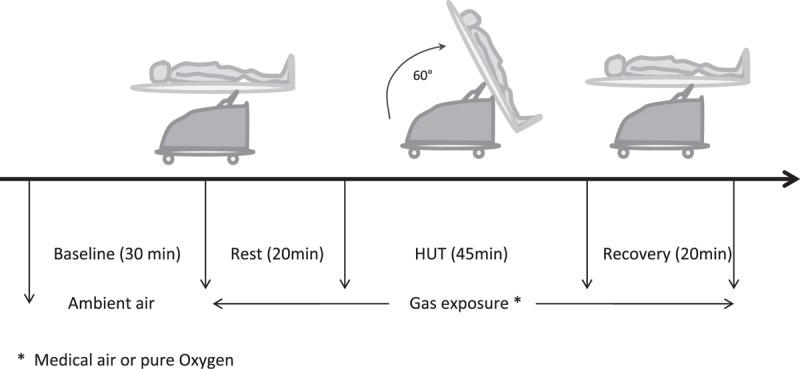
Study protocol for head-up tilt test (HUT).

Each subject passed a screening examination, including physical examination and medical history. All subjects performed regular leisure physical activities. They had no history of serious disease and took no medication for at least 4 weeks before the study. None of the volunteers had a history of syncope or presyncope during the past 2 years. The study took place within 2 weeks of the screening day. Experiments were performed in the morning 3 hours after a light breakfast. An intravenous catheter was inserted before the onset of the tests for blood sampling. The protocol consisted of 2 HUTs, 1 session with those breathing medical air (fraction of inspired oxygen FiO_2_ = 21%) and 1 session with administration of pure oxygen (FiO_2_ = 100%), in a random order (Figure 1). The same technician randomly selected the gas for each subject. This technician was blind to the nature of the gas and did not participate in the investigation. There was a minimum period of 72 hours and a maximum period of 1 week between the 2 sessions. Investigations were performed at the same time of the day for individual subjects. The gases were delivered in a Douglas bag. HUT was performed in a quiet room with regulated ambient temperature (25°C). Before the experiment, the volunteers were instructed to rest for 30 minutes in supine position (baseline), on a motorized tilt table with footplate support (EM/ TTV.08E, Elettromedica s.a.s, Pederobba, TV, Italy). They breathed ambient air. After the measurements at baseline, the experimental session (exposure to medical air or pure oxygen) consisted of a sequence of 3 periods: gas exposure in supine position (rest during 20 min), HUT at 60°C for 45 minutes or until syncope, and recovery in supine position (20 min). Blood samples were collected at the end of each period (baseline, resting period, HUT, and recovery).

Heart rate (HR) was recorded using an analog 3-lead ECG (ECG, BIOPAC, Systems, Inc., CA). BP was continuously monitored using a finger cuff (Finapres model 2300; Ohmeda, Englewood, CO). The arm was supported to maintain the transducer at the heart level. HR and BP data were the average of the recordings during the last 5 minutes of each period (baseline, resting period, HUT, and recovery).

### Adenosine Plasma Concentration Measurement

Blood samples (3 mL) were collected through the catheter and processed as described previously using laboratory-prepared tubes containing 2 mL of cold-stop solution under vacuum.^4^ The sample collection method allowed whole blood to mix quickly with the stop solution to prevent red blood cell uptake and degradation of adenosine.^16,17^ The stop solution was composed of dipyridamole (0.2 mM); ethylene diamine tetracetic acid disodium (Na 2 EDTA 4 mM); erythro-9-(2-hydroxy-3-nonyl adenine [EHNA 5 mM]); alpha, beta-methyleneadenosine 5’ diphosphate (AOPCP 79 mM); 2’ deoxycoformycine 10 μg/mL; and heparin sulfate 1 IU/mL. Adenosine plasma concentration measurement has been described.^4,16,17^ In brief, after collection, samples were put in ice, carried to the laboratory, quickly centrifuged, and deproteinized (perchloric acid 70% 1/10 V). Supernatants were analyzed by high-pressure liquid chromatography (Chrom System, Germany). The intra and interassay coefficients of variation ranged from 3% to 5%.

### Statistical Analysis

All the statistical analyses were performed with R statistical software.^18^ All data (biological and hemodynamic data) recorded during baseline were subtracted from data recorded during rest, tilt, and recovery to produce delta values. Delta values were chosen because the sessions (medical air and pure oxygen) were not performed the same day. Consequently, the hemodynamic data recorded at baseline could be slightly different. Linear mixed model (LMM) analyses were performed with R package “lme4.”^19^ The mixed model approach was chosen over generalized linear model (repeated-measure analysis of variance [ ANOVA]) for the reason that it allows post hoc analysis and is adapted to crossed random effects—here, time and subject.^20^ Least-squares mean calculations and post hoc comparisons were performed with R package “lsmeans,”^21^ with Bonferroni adjustment for *P* value adapted for multiple comparisons.

For BP, HR data, and plasmatic adenosine level, several LMMs were compared, and the most appropriate model (based on lowest Akaike Information Criterion and Bayesian Information Criterion) was the following: “gas type,” “protocol period,” and interaction between “gas type” and “protocol period” for fixed factors; random factors were “subject” and the interaction between “protocol period” and “subject.”

## RESULTS

Fifteen volunteers presented no clinical manifestations during the experiment, whereas 2 experienced presyncope or syncope during HUT.

### Volunteers With Normal Tolerance to HUT

Head-up tilt test was well tolerated by 15 volunteers. Table 1 shows their characteristics.

**TABLE 1:**
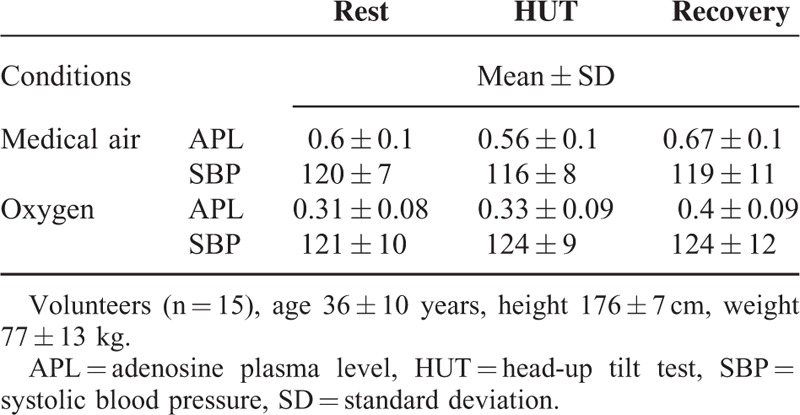
Adenosine Plasma Concentration (μM) and Systolic Blood Pressure (mm Hg) During the 2 Sessions in the Volunteers Without Syncope or Presyncope

### Blood Pressure and Heart Rate

**FIGURE 2:**
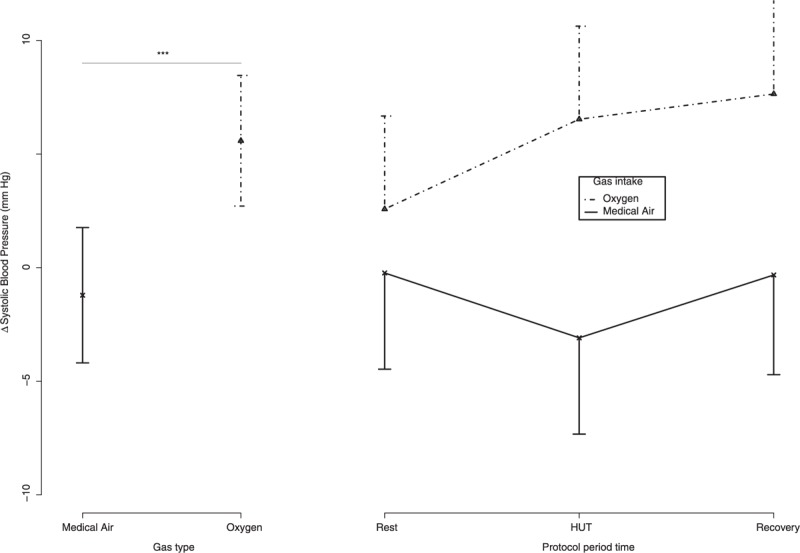
Delta systolic blood pressure (SBP) is represented in 15 healthy volunteers. Plot at the left side showed the delta average and 95% confidence intervals between the gas intake conditions. Plot at the right side showed delta average and 95% confidence intervals during the whole protocol, and between the gas intake conditions. Post hoc differences when needed were expressed as follows: “.” = *P* < 0.10; “∗” = *P* < 0.05; “∗∗” = *P* < 0.01; “∗∗∗” = *P* < 0.001.

Figure 2 reports the delta values in systolic blood pressure (SBP) during the 2 sessions. Delta SBP was higher during hyperoxia (*F*_adjusted_ [1, 41.57] = 8.54, *P* = 0.0056). No difference was found for delta SBP between the protocol periods (*F*_adjuted_ [2, 63.85] = 0.71, *P* = 0.495). No interaction between “gas type” and “protocol period ” was found for delta mean BP (*F*_adjusted_ [2, 40.65] = 1.78, *P* = 0.18).

Delta diastolic blood pressure (DBP) was not significantly different during hyperoxia and medical air (*F*_adjusted_ [1, 41.94] = 1.73, *P* = 0.195). During protocol period (*F*_adjusted_ [2, 64.22] = 3.26, *P* = .0445), delta DBP tended to be higher during HUT than recovery (t.ratio = −3.22, *P* = 0.0097) and rest (t.ratio = −4.42, *P* = 0.0004).

**FIGURE 3:**
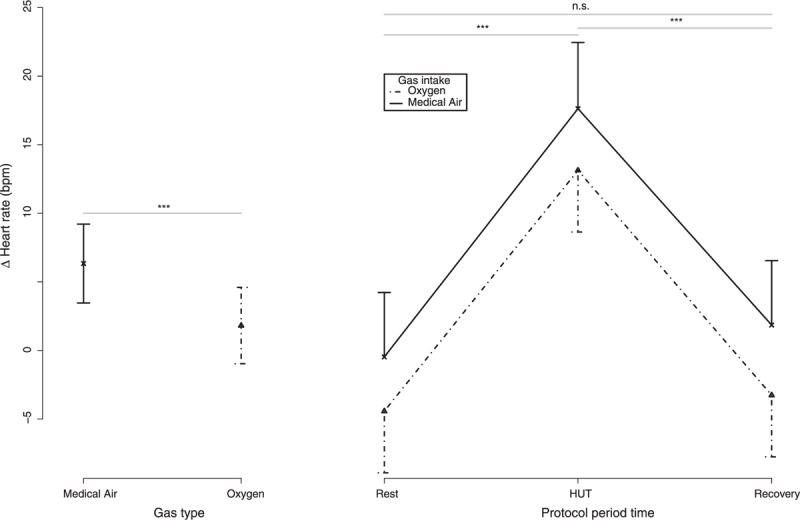
Delta heart rate values are represented in 15 healthy volunteers. Plot at the left side showed the delta average and 95% confidence intervals between the gas intake conditions. Plot at the right side showed delta average and 95% confidence intervals during the whole protocol and between the gas intake conditions. Post hoc differences when needed were expressed as follows: “.” = *P* < 0.10; “∗” = *P* < 0.05; “∗∗” = *P* < 0.01; “∗∗∗” = *P* < 0.001.

Figure 3 reports the delta value in HR during the 2 sessions. The same linear mixed model as above was selected for delta HR. Delta HR was higher (*F*_adjusted_ [1, 37.07] = 6.35, *P* = 0.0161) during medical air exposure. During HUT, delta HR was higher (*F*_adjusted_ [2, 49.53] = 39.58, *P* = 0.0000) than during other periods (rest-HUT: t.ratio = −9.91, *P* = 0.0000; recovery-HUT = −8.94, *P* = 0.0000), but no difference was found between recovery and rest (t.ratio = 0.97, *P* = 0.597). No interaction effect between “gas type” and “protocol period ” was found (*F*_adjusted_ [2, 37.66] = 0.14, *P* = 0.87).

### Plasmatic Adenosine Level

**FIGURE 4:**
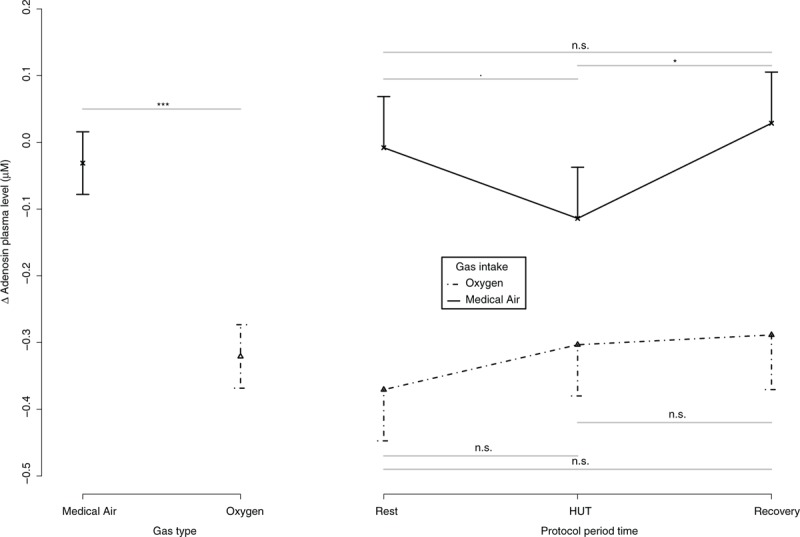
Delta adenosine plasma level (APL) is represented in 15 healthy volunteers. Plot at the left side showed the delta average and 95% confidence intervals between the gas intake conditions. Plot at the right side showed delta average and 95% confidence intervals during the whole protocol and between the gas intake conditions. Post hoc differences when needed were expressed as following: “.” = *P* < 0.10; “∗” = *P* < 0.05; “∗∗” = *P* < 0.01; “∗∗∗” = *P* < 0.001.

Figure 4 reports the changes in delta adenosine plasma concentrations measured in the volunteers during the 2 sessions. The same LMM as that for BP analysis was used for delta APL. Delta APL (*F*_adjusted_ [1,40.40] = 107.16, *P* = 0.0000) was lower during hyperoxia than medical air exposure. Delta APL was lower (*F*_adjusted_ [2, 65.22] = 8.94, *P* = 0.0004) during HUT than in recovery (t.ratio = −3.1, *P* = 0.012) and rest period (t.ratio = −2.34, *P* = 0.07. An interaction was found on delta APL between “gas type” and “protocol period” (*F*_adjusted_ [2, 41.37] = 6.55, *P* = 0.0033). A drop of delta APL was found under medical air exposure during HUT with restoration during recovery (rest-HUT: t.ratio = 3.03, *P* = 0.0532; HUT-recovery: t.ratio = −4.07, *P* = 0.0019; rest-recovery: t.ratio = −1.05, *P* = 1), but not under pure oxygen (rest-HUT: t.ratio = −1.92, *P* = 0.88; HUT-recovery: t.ratio = −0.4, *P* = 1; rest-recovery: t.ratio = −2.24, *P* = 0.43).

### Volunteers With Presyncope or Syncope During HUT

In 2 volunteers, presyncope or syncope appeared during HUT. In these two subjects, the timing of faint argues in favor of vasovagal symptoms rather than orthostatic hypotension.^22^

Volunteer 1 was a healthy student in sport sciences (age 19 years, height 188 cm, weight 73 kg). Under the first test with hyperoxic exposure, the test was well tolerated until the duration initially planned (45 min). During the medical air session, after 8 minutes and 30 seconds in the upright position, the volunteer felt like passing out. A marked decrease was recorded in both HR (from 79 to 41 beats/min) and SBP (from 110 to 60 mm Hg). HUT was immediately terminated and the subject recovered spontaneously.

Volunteer 2 was a well-trained breath-hold diver (age 49 years, height 190 cm, weight 84 kg). He was first submitted to the test with medical air exposure. During this test, a syncope appeared 11 minutes after the passage in upright position. Simultaneously, a sudden drop was recorded in SBP (from 110 to 69 mm Hg) and in HR (from 90 to 56 beats/min). The volunteer experienced a second test under hyperoxia, 1 week later. To compare the APL at the moment of the syncope, the blood sample was collected 11 minutes after the passage in head-up position. No clinical or hemodynamic impairment was observed during this test. Interestingly, a third HUT was performed in this volunteer in the course of another experiment. It was positive with medical air breathing. Table 2 reports the APL in the 2 volunteers with presyncope or syncope during HUT.

**TABLE 2:**
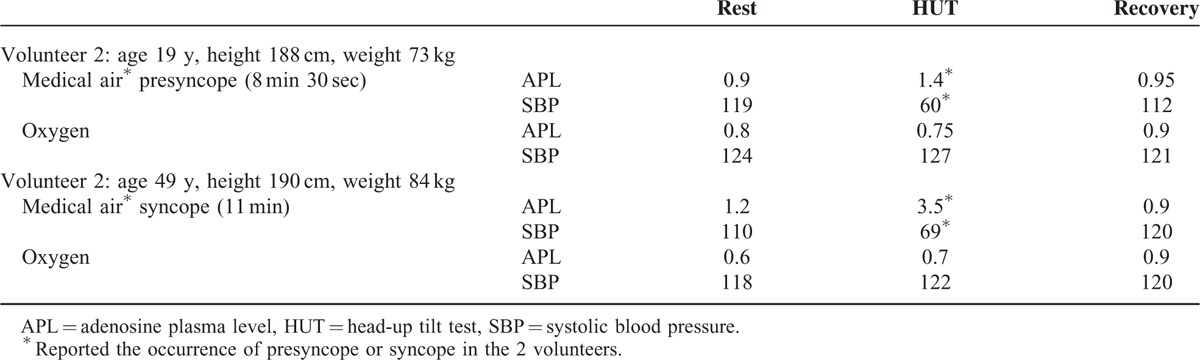
Adenosine Plasma Concentration (in μM) and Systolic Blood Pressure (mm Hg) During the 2 Sessions in the 2 Volunteers Affected by Syncope or Presyncope

## DISCUSSION

The study presents for the first time the impact of hyperoxia on both hemodynamic data and APL in healthy subjects submitted to orthostatic stress. In our study, hyperoxic exposure improved hemodynamic status during HUT.

Systolic BP was increased during hyperoxic session when compared with medical air session in the 15 volunteers with normal tolerance to HUT (Figure 2). A drop in APL was recorded during hyperoxic exposure, both in supine position (rest and recovery) and in orthostatic position (Figure 4). In the 2 volunteers with presyncope or syncope, the administration of pure oxygen improved tolerance to HUT. During the session with medical air breathing, presyncope or syncope occurred in the 2 volunteers at 8 minutes and 30 seconds, and 11 minutes after the passage to the upright position. In contrast, when the subjects breathed pure oxygen, no presyncope or hemodynamic alterations were recorded during the 45 minutes in orthostatic position.

Appropriate cardiovascular adaptation to the passage from supine to standing position includes increase in HR and vasoconstriction. In some subjects, the cardiovascular response is inappropriate and it is well-recognized that fainting and syncope, during a passive transition from supine to upright position, are secondary to a marked decrease in cerebral blood flow secondary to a paradoxical triggering of bradycardia and/or vasodilatation. These abnormal cardiovascular responses are the essential features of reflex syncope and particularly vasovagal syncope.^22,23^ It was reported that the difference in the R-R intervals before and after oxygen inhalation (for 5 min), divided by the difference in the oxygen pressure, evaluated in the venous blood, is lower in neurocardiogenic patients than healthy subjects, suggesting that in these patients, chemoreflex sensitivity is impaired in hyperoxic conditions.^13^ This mechanism may participate in the improvement in orthostatic tolerance of HUT during oxygen exposure. Our results suggest that the improved orthostatic tolerance secondary to oxygen breathing can also be attributed to the vasoconstrictor effect of hyperoxia.^24,25^ The mechanisms explaining the vasoconstriction induced by hyperoxia remain unclear. Some studies have implicated a direct effect of O_2_ or reactive O_2_ species on vascular smooth muscle through an action on calcium channel.^26,27^ Other studies have reported an alteration in endothelial-derived vasoactive agents, such as nitric oxide, Prostaglandins, or endothelin-1.^28–31^ Here we found that hyperoxia was associated with a decrease in APL.

An increase in APL was recorded in upright position in the 2 volunteers with a positive HUT under medical air exposure (Table 2). In contrast, a slight decrease in APL was recorded in volunteers without presyncope or syncope during HUT under medical air exposure (Figure 4). This decrease, previously observed in patients with negative HUT,^4^ may contribute to the cardiovascular adaptive response to orthostatism, probably via a quick adenosine uptake by blood cells.

The extracellular generation of adenosine by nucleotidases is facilitated by the reductions in extracellular pH and in PO_2_. It has been shown that experimental hypoxemia leads to adenosine plasma release, favoring bradycardia and loss of consciousness.^9^ The main cardiovascular changes induced by an increase in APL included decreased HR through A_1_ receptor stimulation and vasodilatation via extraluminal A_2A_ adenosine receptors. These alterations are able to counteract the appropriate cardiovascular adaptation to orthostatic stress in subjects with vasovagal reaction.

### Limitations

Seventeen volunteers were investigated in this study. Two of them experienced presyncope or syncope during HUT. To strengthen our results, a larger sample is needed. Also, further work should study the impact of hyperoxia on the tolerance to orthostatic stress and APL in subjects with vasovagal syncope.

## CONCLUSIONS

In our study, oxygen breathing led to a decrease in APL, when compared with medical air breathing. This decrease was particularly large in upright position (Table 2), and could explain the improvement in both clinical and hemodynamic status during HUT performed under hyperoxia. Consequently, the results of the present study support the implication of adenosine in the inappropriate cardiovascular adaptation during postural stress and argues in the favor of the use of adenosine receptor antagonists in neurocardiogenic syncope.

## Acknowledgments

The authors gratefully acknowledge the volunteers and Mrs Karine Ayme for technical support.

## References

[1] Shryock JC Belardinelli L Adenosine and adenosine receptors in the cardiovascular system: biochemistry, physiology, and pharmacology. *Am J Cardiol* 1997; 79 (12A):2–10. 9223356 10.1016/s0002-9149(97)00256-7

[2] Brignole M Menozzi C Gianfranchi L Neurally mediated syncope detected by carotid-sinus massage and head-up tilt test in sick sinus syndrome. *Am J Cardiol* 1991; 68:1032–1036. 1927916 10.1016/0002-9149(91)90491-3

[3] Kenny Ra Ingram A Bayliss J Head-up tilt: a useful test for investigating unexplained syncope. *Lancet* 1986; 1:1352–1355. 2872472 10.1016/s0140-6736(86)91665-x

[4] Saadjian AY Lévy S Franceschi F Role of endogenous adenosine as a modulator of syncope induced during tilt testing. *Circulation* 2002; 106:569–574. 12147538 10.1161/01.cir.0000023924.66889.4c

[5] Carrega L Saadjian AY Mercier L Increased expression of adenosine A2A receptors in patients with spontaneous and head-up-tilt-induced syncope. *Hear Rhythm* 2007; 4:870–876. 10.1016/j.hrthm.2007.03.002 17599669

[6] Deharo J-C Mechulan A Giorgi R Adenosine plasma level and A2A adenosine receptor expression: correlation with laboratory tests in patients with neurally mediated syncope. *Heart* 2012; 98:855–859. 22581734 10.1136/heartjnl-2011-301411

[7] Guieu R Deharo J-C Ruf J Adenosine and clinical forms of neurally-mediated syncope. *J Am Coll Cardiol* 2015; 66:204–205. 26160639 10.1016/j.jacc.2015.04.066

[8] Grenz A Homann D Eltzschig HK Extracellular adenosine: a safety signal that dampens hypoxia-induced inflammation during ischemia. *Antioxid Redox Signal* 2011; 15:2221–2234. 21126189 10.1089/ars.2010.3665 PMC3166177

[9] Joulia F Coulange M Lemaitre F Plasma adenosine release is associated with bradycardia and transient loss of consciousness during experimental breath-hold diving. *Int J Cardiol* 2013; 168:e138–e141. 24016542 10.1016/j.ijcard.2013.08.053

[10] Rickards CA Newman DG The effect of low-level normobaric hypoxia on orthostatic responses. *Aviat Space Environ Med* 2002; 73:460–465. 12014605

[11] Halliwill JR Minson CT Cardiovagal regulation during combined hypoxic and orthostatic stress: fainters vs. nonfainters. *J Appl Physiol* 19852005; 98:1050–1056. 15531565 10.1152/japplphysiol.00871.2004

[12] Bruzzese L Rostain J-C Née L Effect of hyperoxic and hyperbaric conditions on the adenosinergic pathway and CD26-expression in rat. *J Appl Physiol* 2015; 119:140–147. 25997945 10.1152/japplphysiol.00223.2015

[13] Meyer C Rana OR Saygili E Hyperoxic chemoreflex sensitivity is impaired in patients with neurocardiogenic syncope. *Int J Cardiol* 2010; 142:38–43. 19176256 10.1016/j.ijcard.2008.12.081

[14] Carter JR Lawrence JE Klein JC Menstrual cycle alters sympathetic neural responses to orthostatic stress in young, eumenorrheic women. *AJP Endocrinol Metab* 2009; 297:E85–91. 10.1152/ajpendo.00019.2009 PMC2711656 19401460

[15] Hayashi K Miyachi M Seno N Variations in carotid arterial compliance during the menstrual cycle in young women. *Exp Physiol* 2006; 91:465–472. 16407473 10.1113/expphysiol.2005.032011

[16] Guieu R Sampiéri F Bechis G Use of HPLC to measure circulating adenosine levels in migrainous patients. *Clin Chim Acta* 1994; 227:185–194. 7955415 10.1016/0009-8981(94)90146-5

[17] Bonello L Laine M Kipson N Ticagrelor increases adenosine plasma concentration in patients with an acute coronary syndrome. *J Am Coll Cardiol* 2014; 63:872–877. 24291273 10.1016/j.jacc.2013.09.067

[18] R Core Team. R: A Language and Environment for Statistical Computing. Vienna, Austria; 2015.

[19] Bates D Maechler M Bolker B Walker S {lme4}: Linear mixed-effects models using Eigen and S4. 2014.

[20] Fox J Weisberg S An {R} companion to applied regression. 2nd ed. Thousand Oaks, CA: Sage; 2011.

[21] Lenth RV Hervé M lsmeans: least-squares means. R pack-age version 2.15. http://CRAN.R-project.org/package-lsmeans 2015.

[22] Ricci F De Caterina R Fedorowski A Orthostatic hypotension: epidemiology, prognosis, and treatment. *J Am Coll Cardiol* 2015; 66:848–860. 26271068 10.1016/j.jacc.2015.06.1084

[23] Fu Q Levine BD Pathophysiology of neurally mediated syncope: Role of cardiac output and total peripheral resistance. *Auton Neurosci Basic Clin Elsevier* 2014; 184:24–26. 10.1016/j.autneu.2014.07.004 PMC4139450 25081417

[24] Rossi P Boussuges A Hyperoxia-induced arterial compliance decrease in healthy man. *Clin Physiol Funct Imaging* 2005; 25:10–15. 15659074 10.1111/j.1475-097X.2004.00572.x

[25] Thomson AJ Drummond GB Waring WS Effects of short-term isocapnic hyperoxia and hypoxia on cardiovascular function. *J Appl Physiol* 2006; 101:809–816. 16902069 10.1152/japplphysiol.01185.2005

[26] Jackson WF Arteriolar oxygen reactivity: where is the sensor? *Am J Physiol* 1987; 253 (5 Pt 2):H1120–H1126. 3318502 10.1152/ajpheart.1987.253.5.H1120

[27] Welsh DG Jackson WF Segal SS Oxygen induces electromechanical coupling in arteriolar smooth muscle cells: a role for L-type Ca2 + channels. *Am J Physiol* 1998; 274 (6 Pt 2):H2018–H2024. 9841528 10.1152/ajpheart.1998.274.6.H2018

[28] Demchenko IT Oury TD Crapo JD Regulation of the brain's vascular responses to oxygen. *Circ Res* 2002; 91:1031–1037. 12456489 10.1161/01.res.0000043500.03647.81

[29] Pasgaard T Stankevicius E Jørgensen MM Hyperoxia reduces basal release of nitric oxide and contracts porcine coronary arteries. *Acta Physiol* 2007; 191:285–296. 10.1111/j.1748-1716.2007.01745.x 17784906

[30] Rubanyi GM Vanhoutte PM Superoxide anions and hyperoxia inactivate endothelium-derived relaxing factor. *Am J Physiol* 1986; 250 (5 Pt 2):H822–H827. 3010744 10.1152/ajpheart.1986.250.5.H822

[31] Dallinger S Dorner GT Wenzel R Endothelin-1 contributes to hyperoxia-induced vasoconstriction in the human retina. *Investig Ophthalmol Vis Sci* 2000; 41:864–869. 10711705

